# Population pharmacokinetics of high-dose methotrexate in patients with primary central nervous system lymphoma

**DOI:** 10.3389/fphar.2025.1578033

**Published:** 2025-05-19

**Authors:** Shifeng Wei, Sitian Zhang, Dan Wang, Dongjie Zhang, Qian Lu, Jiayi Mo, Zhilin Yang, Leyi Guan, Yingjun He, Zhigang Zhao, Shenghui Mei

**Affiliations:** ^1^ Department of Pharmacy, Beijing Tiantan Hospital, Capital Medical University, Beijing, China; ^2^ Department of Clinical Pharmacology, College of Pharmaceutical Sciences, Capital Medical University, Beijing, China; ^3^ School of Basic Medical Sciences, Capital Medical University, Beijing, China; ^4^ Department of Pharmacy, Bijie Maternal and Child Health Hospital, Bijie, Guizhou, China

**Keywords:** methotrexate, primary central nervous system lymphoma, population pharmacokinetic model, nonlinear mixed-effects modeling, estimated glomerular filtration rate, blood urea nitrogen, alanine aminotransferase, total protein

## Abstract

**Objective:**

Methotrexate (MTX) serves as a cornerstone therapy for primary central nervous system lymphoma (PCNSL). However, the considerable intra- and inter-individual variability in its pharmacokinetic and therapeutic efficacy poses significant challenges to clinical application. This study aims to employ population pharmacokinetic (PPK) models to investigate the pharmacokinetics of MTX in Chinese patients with PCNSL, thereby facilitating personalized therapeutic strategies for these patients.

**Method:**

A retrospective dataset comprising 6074 MTX plasma concentrations from 752 adult patients with PCNSL receiving high-dose methotrexate (HD-MTX) therapy was employed to construct the PPK model, utilizing the nonlinear mixed-effects modeling approach. The pharmacokinetics of MTX were characterized using a three-compartment model in conjunction with a proportional residual model. Covariate effects on model parameters were evaluated using forward addition and backward elimination approaches. Model performance was assessed through goodness-of-fit, bootstrap analysis, and visual predictive checks.

**Result:**

In the final PPK models, the estimated glomerular filtration rate (eGFR), blood urea nitrogen (BUN), alanine aminotransferase (ALT), and a combined genotype of *ABCC-ABCG-ADORA2A* were identified as significant covariates impacting the clearance (CL) of MTX. Additionally, total protein (TP) was found to be a significant covariate influencing inter-compartmental clearance (Q). The relationship between pharmacokinetic parameters and covariates was quantified as follows: CL (L/h) = 8.45×(eGFR⁄101.8)^0.67^×(BUN⁄4.6)^−0.08^×(ALT⁄25)^0.03^×a (a = 0.91 for gene-model if *ABCC-ABCG-ADORA2A* mutation, otherwise a = 1); Q_1_ (L/h) = 0.04×(TP⁄58)^b^ (b = −1.68 for nongene-model and b = −1.72 for gene-model). Bootstrap analysis and visual predictive checks demonstrated the stability and adequate predictive capacity of the final PPK models.

**Conclusion:**

In managing HD-MTX therapy for PCNSL patients, it is essential to consider pharmacokinetic factors such as eGFR, BUN, ALT, TP, and genetic polymorphisms. The PPK models developed will aid in optimizing and personalizing HD-MTX treatment for PCNSL patients.

## 1 Introduction

Primary central nervous system lymphoma (PCNSL) is a rare and highly malignant form of non-Hodgkin lymphoma, accounting for approximately 4% of newly diagnosed malignant brain tumors (Löw et al., 2018; Schaff and Grommes, 2022). High-dose methotrexate (HD-MTX) based chemotherapy is currently considered the first-line treatment for PCNSL (Calimeri et al., 2021; Hoang-Xuan et al., 2023; Martinez-Calle et al., 2022; Morales-Martinez et al., 2021; Roth and Hoang-Xuan, 2014; Yang H. et al., 2020). Methotrexate (MTX), an antineoplastic folate antagonist, primarily inhibits dihydrofolate reductase, thereby obstructing the biosynthesis of purine and pyrimidine nucleotides, essential for DNA synthesis (Joerger et al., 2012; Csordas et al., 2013). However, MTX has a narrow therapeutic window and exhibits significant interindividual variability in its pharmacokinetics (Dervieux et al., 2004).

HD-MTX, defined as a dose exceeding 500 mg/m^2^, is associated with significant toxicity that may necessitate the interruption or discontinuation of chemotherapy (Gao et al., 2021). Such interruptions can compromise the efficacy of the antitumor treatment and increase the risk of disease relapse (Howard et al., 2016). Despite the implementation of supportive care measures during HD-MTX administration, such as folate supplementation, intravenous hydration, and urine alkalinization, acute kidney injury (AKI) occurs in 2%–12% of patients (Widemann et al., 2010). AKI impairs renal clearance of MTX, leading to drug accumulation and subsequent adverse effects, including myelosuppression, mucositis, hepatotoxicity, and even multi-organ failure (Widemann et al., 2010).

Pharmacokinetic-guided dose adjustment and leucovorin rescue are critical components of individualized MTX therapy. Population pharmacokinetic (PPK) approaches facilitate the quantification and analysis of covariate effects and the integration of sparse pharmacokinetics data, thus gaining widespread application in individualized treatment (Williams and Ette, 2000). Current research indicates that various factors influence MTX pharmacokinetics. Body weight significantly affects the volume of distribution of MTX (Green et al., 2006). Approximately 50% of MTX is bound to proteins, with albumin, globulin, total protein (TP), and concomitant medications (e.g., proton pump inhibitors, nonsteroidal anti-inflammatory drugs, salicylates, levetiracetam, dexamethasone, and penicillin) potentially altering its pharmacokinetics (Joerger et al., 2006; Kim et al., 2012; Panetta et al., 2020; Shi et al., 2020). MTX is primarily eliminated through renal, with serum creatinine (Scr), creatinine clearance (CLcr), and estimated glomerular filtration rate (eGFR) commonly used to adjust MTX clearance (Gao et al., 2021; Joerger et al., 2006; Kim et al., 2012; Panetta et al., 2020; Shi et al., 2020; Batey et al., 2002; Faltaos et al., 2006; Fukuhara et al., 2008; Min et al., 2009; Johansson et al., 2011; Simon et al., 2013; Zhang et al., 2015; Nader et al., 2017; Mei et al., 2018a; Hui et al., 2019; Kawakatsu et al., 2019; Pai et al., 2020; Taylor et al., 2020; Yang L. et al., 2020; Mao et al., 2022; Arshad et al., 2021; Isono et al., 2021). Age, sex, body weight, liver function, hematocrit, and urine output also influence MTX clearance (Batey et al., 2002; Faltaos et al., 2006; Min et al., 2009; Johansson et al., 2011; Zhang et al., 2015; Nader et al., 2017; Mei et al., 2018a; Aumente et al., 2006; Colom et al., 2009; Zhang et al., 2010; Faganel et al., 2011; Lui et al., 2018). Furthermore, efflux and uptake transporters, along with their associated genetic polymorphisms such as *SLCO1B1*, *ABCC2*, *ABCB1*, *ABCG2*, and *MTHFR*, play critical roles in modulating MTX pharmacokinetics, potentially resulting in significant variability in pharmacokinetic parameters (Kim et al., 2012; Simon et al., 2013; Faganel et al., 2011; Lui et al., 2018; Schulte et al., 2021; Wang et al., 2019).

Although several PPK models for HD-MTX have been developed, their reliability across different centers remains insufficient (Mao et al., 2022; Yang et al., 2023). Given the heterogeneity in disease types, treatment regimens, and demographic data among patients, further PPK studies encompassing diverse populations are necessary to refine these models. This study aims to develop PPK models for HD-MTX in Chinese patients with PCNSL and to identify covariates that may influence MTX pharmacokinetic parameters.

## 2 Materials and methods

### 2.1 Study design

This study was a retrospective analysis conducted under the rigorous review and approval of the Ethics Committee of Beijing Tiantan Hospital, Capital Medical University, in strict adherence to the ethical guidelines of the Declaration of Helsinki (ID: KY 2019-072-02). The study population comprised hospitalized patients with PCNSL who received HD-MTX treatment at the hospital between September 2016 and August 2023.

The inclusion criteria for the study were: (1) receipt of intravenous MTX therapy; (2) methotrexate dosage of ≥0.5 g/m^2^; (3) undergoing therapeutic drug monitoring during treatment with at least one MTX concentration measurement obtained; (4) relevant information on the start and end times of MTX administration and sampling times was available. Exclusion criteria encompassed: (1) age <18 years; (2) incomplete data records; (3) methotrexate concentrations below the lower limit of quantification (0.002 μmol/L).

The study systematically documented patient demographics information (including sex, age, height, weight, body mass index [BMI], body surface area [BSA]) and detailed administration (including dosage, start and end times of infusion, sampling times). Additionally, the study collected data on renal function indicators (Scr, CLcr, eGFR, blood urea nitrogen [BUN]), liver function indicators (alanine aminotransferase [ALT], aspartate aminotransferase [AST], TP, albumin, and globulin), and hematological parameters (white blood cell count, red blood cell count, hematocrit, hemoglobin). Biochemical analyses were performed using the Hitachi LABOSPECT 008 AS automated biochemical analyzer. In clinical practice, hepatic and renal function are routinely evaluated prior to MTX administration and monitored daily for at least three consecutive days thereafter, with additional assessments performed as clinically indicated. For missing laboratory values on a given day, the nearest available results—typically within 1–3 days before or after—were used as substitutes. Values were considered missing and excluded from analysis if no relevant laboratory results were available within a 7-day window before or after the target date. BSA calculations employed the Stevenson formula (Equation 1), while CLcr and eGFR were computed using the Cockcroft-Gault formula (Equation 2) and the 2021 CKD-EPI formula (Equation 3), respectively (Cockcroft and Gault, 1976; Inker et al., 2021). Concurrent co-administration of medications with potential influence on MTX pharmacokinetics, including proton pump inhibitors, nonsteroidal anti-inflammatory drugs, salicylates, penicillin, and levetiracetam, was systematically documented. Concurrent co-administration of medications was defined as the use of the drug within 24 h preceding the plasma concentration measurement. Rigorous data validation conducted by two independent researchers ensured the accuracy and reliability throughout the dataset. Statistical analyses were performed using SPSS, version 27.0 (IBM Corp). Descriptive statistics were reported as medians with ranges (minimum–maximum).

$$\text{BSA }\left(m2\right)=0.0061\times H+0.0128\times \text{BW}-0.1529$$ (1)

$$\text{CLcr }\left(\text{mL}/\min\right)=\frac{\left(140-\text{age}\right)\times \text{BW}}{\text{Scr}\times 72}\;\left(\times 0.85\text{ if female}\right)$$ (2)

$$\text{eGFR}=142\times {\left(\frac{\text{Scr}}{A}\right)}^{B}\times 0.9938^{\text{Age}}\times C;\;\left(\text{If female}:C=1.012;\text{when Scr}\leq 0.7\text{ mg}/\text{dL},A=0.7,B=-0.241;\text{when Scr}>0.7\text{ mg}/\text{dL},A=0.7,B=-1.2.\text{If male}:C=1;\text{when Scr}\leq 0.9\text{ mg}/\text{dL},A=0.9,B=-0.302;\text{when Scr}>0.9\text{ mg}/\text{dL},A=0.9,B=-1.2\right)$$ (3)

### 2.2 Methotrexate treatment regimens and therapeutic drug monitoring

In this study, the most common MTX-based regimens involved combination therapy with rituximab or cytarabine. Additional concomitant agents included etoposide, ifosfamide, temozolomide, doxorubicin, thiotepa, ibrutinib, orelabrutinib, zanubrutinib, and lenalidomide. MTX was administered via intravenous infusion at a dose of 3.5 g/m^2^, with a median infusion duration of 3.1 h. Each patient received a median of four MTX infusions (range, 1–34). Administration protocols included either a single infusion or a divided regimen in which 2 g was infused over 0.5 h, followed by the remaining dose over the subsequent 2.5 h. Blood samples were routinely collected on the morning of the second day after administration (approximately 4–5 AM), with additional sampling on the third and fourth mornings as clinically indicated. If MTX plasma concentrations remained elevated, further measurements were performed. Leucovorin rescue was initiated 6 h post-infusion, with a typical total dose of 500 mg (approximately 10%–15% of the MTX dose). The initial two doses consisted of 100 mg each, followed by six doses of 50 mg administered every 6 h. In cases of delayed MTX clearance, the dosing interval was shortened to every 3 h or the dose was increased based on plasma concentration. For example, if the 24-h MTX concentration was ≥100 μmol/L, leucovorin was administered at 1,000 mg/m^2^ every 6 h. For concentrations between 10 and 100 μmol/L, leucovorin was given at 100 mg/m^2^ every 6 or 3 h, depending on clinical judgment.

This study employed ultra-high performance liquid chromatography-tandem mass spectrometry to measure total plasma concentrations of MTX, encompassing both the protein-bound and free fractions. The ion pair selected was m/z 455.2 > 308.2, employing a CMS9030 chromatographic column (Rephi, 2.1 × 50 mm, 3 μm). Methanol was used for protein precipitation during sample preparation, followed by gradient elution with a methanol and 10% ammonium acetate solution. Chromatographic separation was achieved in 2.6 min at a flow rate of 0.4 mL/min, with each injection volume set at 2 µL and a total run time of 3 min. The method demonstrated excellent linearity across MTX concentrations ranging from 0.002 to 2 μmol/L. The intra-day and inter-day inaccuracies ranged from −5.50% to 10.93%, with imprecision remaining below 9.20%. The recovery, normalized using the internal standard MTX-D3, along with the matrix factor, was consistent across all four quality control levels (Mei et al., 2018b).

### 2.3 Genotype identification

Drawing on prior studies and the Pharm GKB database (https://www.pharmgkb.org), we selected 29 single nucleotide polymorphisms with a mutation frequency greater than 0.05 in the Chinese population that are potentially associated with the pharmacokinetics and pharmacodynamics of MTX (Zhao et al., 2021). Details of all variants, including *MTHFR, MTR, ATIC, ABCG2, MTRR, ABCB1, ABCC2, ABCC4, MTHFD1, SLCO1B1, SLC28A2, TYMS*, and *SLC19A1*, are listed in Supplementary Appendix SA1. DNA from patients was extracted from peripheral blood leukocytes using the QIAamp DNA Microbiome Kit (Qiagen, Hilden, Germany) following standard procedures. Genotyping was conducted using the MassARRAY method (Sequenom, United States) at Beijing Bio Miao Biotechnology Co., Ltd. Minor allele frequency, genotype distribution, and Hardy-Weinberg equilibrium (P value and χ^2^ test) for all selected alleles were assessed using PLINK software (version 1.90; Shaun Purcell, Boston, United States).

### 2.4 Grouping and combination of variants

In this study, each selected genetic variant was classified into two or three groups based on genotype, with the number of groups increasing in accordance with decreasing MTX clearance (Table 1). For example, in the three-category grouping, the *ABCG2* rs2231142 G>T variant, with the T allele, was associated with decreased MTX clearance. As a result, the genotypes GG, GT, and TT were categorized into groups 1, 2, and 3, respectively. In cases where individual genetic variants did not significantly influence MTX clearance, the potential cumulative effect of multiple variants was considered by combining them. The combined variant was generated by summing the three categorical group numbers of any two or three significant variants and was then classified into two groups according to predefined combination rules (Table 2). The new group number also increased as MTX clearance decreased. For the combined variant, a binary grouping was employed, with 1 representing individuals without mutations and 2 representing those with mutations.

**TABLE 1:** Grouping rules for variants.

Group type	Methotrexate clearance changing by variants	Wild-type homozygote	Heterozygote	Variant homozygote
Three groups	Increased	3	2	1
	Decreased	1	2	3
Two groups, rule 1	Increased	2	2	1
	Decreased	1	1	2
Two groups, rule 2	Increased	2	1	1
	Decreased	1	2	2

**TABLE 2:** Combination rules for variants.

Number of combined variants	Combined new group type	Summation of new group numbers	Combined new group number
2	Two groups, rule 1	2	1
		3–6	2
	Two groups, rule 2	2–3	1
		4–6	2
	Two groups, rule 3	2–4	1
		5–6	2
3	Two groups, rule 1	3–5	1
		6–9	2
	Two groups, rule 2	3–6	1
		7–9	2
	Two groups, rule 3	3–7	1
		8–9	2

### 2.5 Development of population pharmacokinetic model

The study employed Phoenix^®^ NLME software (version 8.3; Certara, St. Louis, Missouri) to develop a PPK model using the nonlinear mixed-effects approach. Parameter estimation was performed using the first-order conditional estimation extended least squares method. Model comparisons were based on objective function value (OFV), Akaike information criterion (AIC), and Bayesian information criterion (BIC). Model stability and predictive performance were assessed through bootstrap validation and visual predictive check (VPC).

#### 2.5.1 Base model

**FIGURE 1:**
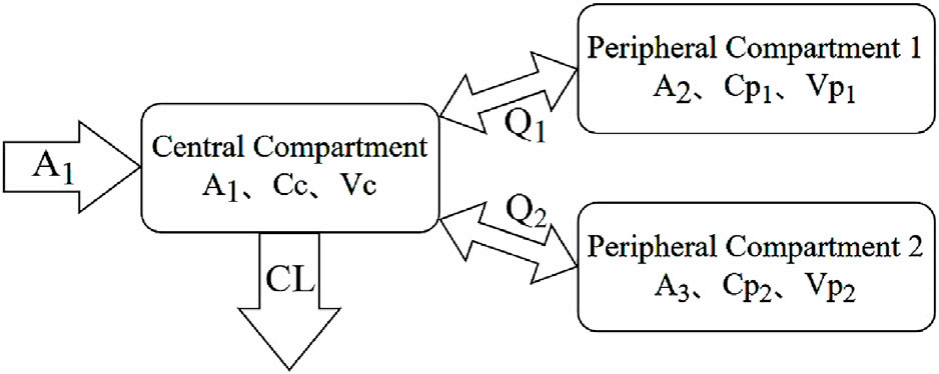
The schematic pharmacokinetic model of methotrexate.

Each MTX administration was treated as an independent event, as the dosing intervals exceeded 5 elimination half-lives, allowing for near-complete drug clearance between doses. The pharmacokinetics of MTX in patients were characterized using a first-order elimination three-compartment model. Model parameters comprised central compartment clearance (CL), apparent volume of the central compartment (Vc), apparent volume of the peripheral compartments (Vp_1_ and Vp_2_), and inter-compartmental clearance (Q_1_ and Q_2_). The model structure is depicted in Figure 1, with the corresponding mathematical expressions provided in Equations 4–9. Inter-individual variability (IIV, η) in pharmacokinetic parameters was assessed using an exponential error model, as indicated in Equation 10. Multiple error models were evaluated, including a proportional error model, an exponential model, an additive model, and a combined additive-proportional error model. The results indicated that the proportional error model provided the best fit. Consequently, residual variability (ε) in MTX concentrations was described using a proportional model, as shown in Equation 11. It was assumed that the random variables η and ε followed a normal distribution with a mean of 0 and variances ω^2^ and σ^2^, respectively. In these equations, θ_TV_ represents the typical population value of the pharmacokinetic parameters, while Cobs and Cpred denote the observed and predicted concentrations, respectively.

$${dA}_{1}/dt=-CL\times C_{c}-Q_{1}\times \left(C_{c}-C_{p1}\right)-Q_{2}\times \left(C_{c}-C_{p2}\right)$$ (4)

$${dA}_{2}/dt=Q_{1}\times \left(C_{c}-C_{p1}\right)$$ (5)

$${dA}_{3}/dt=Q_{2}\times \left(C_{c}-C_{p2}\right)$$ (6)

$$C_{c}=A_{1}/V_{c}$$ (7)

$$C_{p1}=A_{2}/V_{p1}$$ (8)

$$C_{p2}=A_{3}/V_{p2}$$ (9)

$$\theta =\theta_{TV}\times e^{\eta }$$ (10)

$$C_{obs}=C_{pred}\times \left(1+\varepsilon \right)$$ (11)

#### 2.5.2 Covariate model

Following the establishment of the base model, the impact of covariates on MTX pharmacokinetic variability was further examined. All continuous covariates were standardized to their median values. The forward addition and backward elimination approach was utilized to evaluate the effects of covariates on MTX pharmacokinetic parameters. During the forward addition, a change in the objective function value (ΔOFV) exceeding 3.83 (*P* < 0.05, df = 1) indicated a significant impact of the covariate on the parameter, warranting its inclusion in the model. Conversely, in the backward elimination phase, a ΔOFV greater than 6.64 (*P* < 0.01, df = 1) signified a significant covariate effect, necessitating its retention in the model; otherwise, the covariate was excluded.

#### 2.5.3 Goodness-of-fit and model evaluation

Scatter plots were employed to evaluate the goodness-of-fit for both the base and final models. These plots included observed concentrations versus population predicted concentrations (PRED), observed concentrations versus individual predicted concentrations (IPRED), and conditional weighted residuals (CWRES) versus PRED and time after dose (TAD). To evaluate the final model’s stability and predictive performance, we conducted bootstrap and VPC analyses. Owing to the large sample size and the complexity of the model structure, conducting a 1,000 bootstrap analysis was computationally intensive and time-consuming. Therefore, 200 bootstrap replicates were performed to estimate the median and 95% confidence intervals (95%CI, 2.5%–97.5%) for the model parameters. These bootstrap-derived estimates were then compared with the corresponding parameter values obtained from the final model to assess estimation stability. During the VPC analysis, we performed 1,000 Monte Carlo simulations to calculate the 10th, 50th, and 90th percentiles of the simulated outcomes, along with their corresponding 80% prediction intervals, which were compared with the distribution of observed values.

## 3 Results

### 3.1 Demographic data and genotyping of enrolled patients

The study cohort consisted of 752 adult Chinese patients, including 420 males and 332 females, from whom a total of 6074 MTX plasma concentration samples were collected. According to the MTX drug label, delayed elimination is defined as a serum MTX concentration exceeding 50 μmol/L at 24 h, 5 μmol/L at 48 h, 0.2 μmol/L at 72 h, or 0.05 μmol/L at 96 h following administration. Based on these criteria, at least 17.4% of patients in this cohort exhibited evidence of delayed MTX clearance. The median age was 57.45 years, and the median weight was 68 kg. Omeprazole and levetiracetam were concurrently used by 41.03% and 35.65% of patients, respectively. Comprehensive patient demographics, laboratory findings, and concomitant medications are detailed in Table 3. The temporal distributions of MTX plasma concentrations and eGFR are shown in Supplementary Appendices SA2, SA3, respectively. A total of 29 genetic variants were analyzed, with detailed information provided in Supplementary Appendix SA1. Except for rs10760502, rs11045879, rs2413775, and rs3758149, the allele frequencies of the remaining variants were consistent with Hardy-Weinberg equilibrium (*P* > 0.05).

**TABLE 3:** Characteristics of patients in the population pharmacokinetic model.

Variable	Median (range) and N (%)
No. of. subjects	752
No. of. concentration sample	6,074
Sex (Male/Female)	420 (55.9%)/332 (44.1%)
Age (years)	57.445 (18.12–86.65)
Body weight (kg)	68 (30–115)
Height (cm)	167 (146–192)
BMI (kg/m2)	24.24 (12.98–37.55)
BSA (m2)	1.73 (1.16–2.39)
MTX concentration (umol/L)	0.116 (0.002–752)
Serum creatinine (μmol/L)	64.6 (24.8–641.7)
CLCR (mL/min)	100.1 (5.9–361.8)
eGFR (mL/min/1.73m2)	101.8 (5.4–162.9)
Blood urea nitrogen (mmol/L)	4.6 (0.5–19)
Aspartate aminotransferase (U/L)	20.4 (5–1915.2)
Alanine aminotransferase (U/L)	25 (2.2–1,141.7)
Albumin (g/L)	37.5 (19.9–51.8)
Globulin (g/L)	24.3 (5.9–60.3)
Total protein (g/L)	61.8 (27.4–95.7)
RBC (10^12^/L)	3.88 (1.64–7.93)
WBC (10^9^/L)	6.91 (0.17–120.46)
NEUT (10^9^/L)	5.02 (0.01–84.56)
GR (%)	74.4 (1.8–99.1)
HCT (%)	35.9 (15.3–66.1)
HGB (g/L)	121 (38–202)
MCH (pg)	31.3 (9.3–45)
MCHC (g/L)	340 (256–454)
MCV (fL)	92 (59.1–116.4)
PLT (10^9^/L)	217 (7–902)
Co-medications^a^	
Omeprazole	2,494 (41.03%)
Ilaprazole	381 (6.27%)
Furosemide	962 (15.82%)
Torasemide	1,514 (24.91%)
Bumetanide	979 (16.10%)
Levetiracetam	2,167 (35.65%)
NSAIDs	213 (3.51%)

^a^ Co-medications were a count of methotrexate concentration samples, rather than the number of patients.

### 3.2 Development of population pharmacokinetic model

Incorporating genetic variants into the model allows for the identification of potential associations between gene variations and drug metabolism, providing further insights into personalized therapy. However, given the limited routine implementation of genetic testing in clinical practice, a model without genetic factors was also developed to enhance clinical applicability. Consequently, this study established two final population pharmacokinetic models for MTX: one integrating genetic polymorphisms (gene-model) and the other excluding genetic factors (nongene-model). The study findings reveal that eGFR exerted the most pronounced influence on MTX clearance dynamics. Higher eGFR were associated with accelerated MTX clearance (ΔOFV = −471.89, *P* < 0.05). Moreover, BUN, ALT and the ABCC-ABCG-ADORA2A gene polymorphism were found to impact MTX clearance (ΔOFV = −62.08, −12.51 and −9.75, respectively, *P* < 0.05), while TP significantly influenced inter-compartmental clearance (ΔOFV = −30.02, *P* < 0.05). Covariates other than these did not demonstrate statistically significant effects and were therefore not incorporated into the model. The detailed process of model development is outlined in Table 4, and the quantitative relationships between the final model parameters and covariates are described by Equations 12–19.

**TABLE 4:** Results in the model development procedure of two final models.

Model no.	Model description	OFV	∆OFV	*P* Value
Forward addition				
1	Base model	17.69		
2	Add eGFR on CL in model 1	−454.20	−471.89	<0.05
3	Add TP on Q in model 2	−511.01	−56.81	<0.05
4	Add BUN on CL in model 3	−536.21	−25.20	<0.05
5 (Nongene-model)	Add ALT on CL in model 4	−554.21	−18.01	<0.05
6 (Gene-model)	Add GEN on CL in model 5	−564.16	−9.95	<0.05
Backward elimination				
7	Remove eGFR on CL in model 5 (Nongene-model)	−114.36	439.85	<0.01
8	Remove TP on Q in model 5 (Nongene-model)	−502.82	51.40	<0.01
9	Remove BUN on CL in model 5 (Nongene-model)	−524.16	30.06	<0.01
10	Remove ALT on CL in model 5 (Nongene-model)	−536.21	18.01	<0.01
11	Remove eGFR on CL in model 6 (Gene-model)	−104.88	459.29	<0.01
12	Remove TP on Q in model 6 (Gene-model)	−514.67	49.49	<0.01
13	Remove BUN on CL in model 6 (Gene-model)	−534.41	29.75	<0.01
14	Remove ALT on CL in model 6 (Gene-model)	−546.25	17.91	<0.01
15	Remove GEN on CL in model 6 (Gene-model)	−554.21	9.95	<0.01

Nongene-Model:

$$CL\;\left(L/h\right)=8.2\times {\left(eGFR/101.8\right)}^{0.67}\times {\left(BUN/4.6\right)}^{-0.08}\times {\left(ALT/25\right)}^{0.03}$$ (12)

$$Q_{1}\;\left(L/h\right)=0.04\times {\left(TP/58\right)}^{-1.68}$$ (13)

Gene-Model:

$$CL\;\left(L/h\right)=8.45\times {\left(eGFR/101.8\right)}^{0.67}\times {\left(BUN/4.6\right)}^{-0.08}\times {\left(ALT/25\right)}^{0.03}\times 0.91\left(\text{If }ABCC-ABCG-ADORA2A\text{ mutation}\right)$$ (14)

$$Q_{1}\;\left(L/h\right)=0.04\times {\left(TP/58\right)}^{-1.72}$$ (15)

$$Q_{2}\;\left(L/h\right)=0.09$$ (16)

$$Vc\;\left(L\right)=33.3$$ (17)

$$V_{p1}\;\left(L\right)=17.9$$ (18)

$$V_{p2}\;\left(L\right)=1.14$$ (19)

In the equations, 8.2 and 8.45 represent the typical values of CL (L/h) for the nongene-model and gene-model, respectively. The values 0.04, 0.09, 33.3, 17.9, and 1.14 correspond to the typical population estimates for Q_1_ (L/h), Q_2_ (L/h), Vc (L), V_p1_ (L), and V_p2_ (L), respectively, and are consistent across both models. The relationship coefficients between CL and eGFR, BUN, and ALT are 0.67, −0.08, and 0.03, respectively. For Q_1_, the coefficients with TP are −1.68 and −1.72 for the nongene and gene models, respectively. Additionally, the gene *ABCC-ABCG-ADORA2A* refers to a composite genotype encompassing three specific variants: *ABCC4* rs2274407 (T>G), *ABCG2* rs2231142 (G>T), and *ADORA2A* rs2298383 (C>T). Patients were identified as mutation carriers of the *ABCC-ABCG-ADORA2A* genotype if they exhibited more than three nucleotide mutations among these variants. Detailed estimates for the base model, final pharmacokinetic parameters, relative standard errors, 95% confidence intervals (CI), inter-individual variability, residual variability, and Bootstrap analysis results are presented in Table 5.

**TABLE 5:** Parameter estimates and Bootstrap results of methotrexate population pharmacokinetic model.

Parameter	Final nongene-model		Bootstrap nongene-model		Final gene-model		Bootstrap gene-model	
	Estimate (%RSE)	95% CI	Median (%RSE)	95% CI	Estimate (%RSE)	95% CI	Median (%RSE)	95% CI
CL (L/h)	8.2 (2.83)	(7.75, 8.66)	8.21 (7.14)	(7.15, 9.39)	8.45 (2.98)	(7.95, 8.94)	8.50 (6.62)	(7.38, 9.71)
Q_1_ (L/h)	0.04 (8.13)	(0.03, 0.05)	0.04 (13.94)	(0.03, 0.05)	0.04 (8.14)	(0.03, 0.05)	0.04 (13.81)	(0.03, 0.05)
Q_2_ (L/h)	0.09 (5.18)	(0.08, 0.10)	0.09 (10.67)	(0.07, 0.11)	0.09 (5.14)	(0.08, 0.10)	0.09 (10.36)	(0.07, 0.11)
Vc (L)	33.39 (3.51)	(31.09, 35.69)	33.5 (9.82)	(27.53, 39.82)	33.29 (3.50)	(31.00, 35.57)	33.52 (9.15)	(27.7, 40.44)
V_P1_ (L)	17.9 (11.52)	(13.86, 21.94)	18.39 (20.81)	(12.58, 28.03)	17.85 (11.48)	(13.84, 21.87)	18.32 (24.57)	(11.77, 30.67)
V _P2_ (L)	1.14 (4.40)	(1.04, 1.23)	1.14 (9.04)	(0.93, 1.36)	1.14 (4.36)	(1.04, 1.24)	1.15 (8.94)	(0.96, 1.37)
*θ*_*eGFR*_	0.67 (2.10)	(0.65, 0.70)	0.67 (11.10)	(0.54, 0.84)	0.67 (2.10)	(0.64, 0.70)	0.67 (11.04)	(0.56, 0.85)
*Θ*_*BUN*_	−0.08 (10.23)	(-0.09, −0.06)	−0.08 (38.04)	(-0.14, −0.03)	−0.08 (10.20)	(-0.09, −0.06)	−0.07 (38.35)	(-0.13, −0.02)
*θ*_*ALT*_	0.03 (14.47)	(0.02, 0.03)	0.03 (44.34)	(0, 0.05)	0.03 (14.23)	(0.02, 0.03)	0.03 (42.92)	(0.00, 0.05)
*θ*_*TP*_	−1.68 (8.3)	(-1.96, −1.41)	−1.64 (−37.89)	(-2.97, −0.5)	−1.72 (8.12)	(-1.99, −1.44)	−1.73 (43.04)	(-3.30, −0.31)
*θ*_*ABCC4-ABCG2-ADORA2A*_	-	-	-	-	−0.09 (−30.63)	(-0.14, −0.03)	−0.08 (28.65)	(-0.13, −0.04)
IIV_CL_ (CV%)	27.3 (1.86)	-	27.2 (4.79)	-	27.1 (1.88)	-	26.81 (4.04)	-
IIV_Q1_ (CV%)	98.91 (9.43)	-	100.24 (15.25)	-	99.01 (9.47)	-	99.92 (14.97)	-
IIV_Vc_ (CV%)	20.95 (5.77)	-	21.42 (7.27)	-	20.27 (5.91)	-	21.26 (6.47)	-
IIV_Vp1_ (CV%)	78.35 (25.38)	-	78.73 (25.15)	-	78.1 (25.46)	-	79.68 (25.05)	-
IIV_Vp2_ (CV%)	26.95 (5.22)	-	27.11 (5.51)	-	27.2 (5.23)	-	27.34 (5.52)	-
σ (proportional)	73.79 (1.07)	(72.24, 75.35)	73.49 (2.96)	(69.49, 77.89)	73.79 (1.07)	(72.24, 75.34)	73.75 (3.01)	(69.66, 78.42)

### 3.3 Goodness-of-fit and model evaluation

**FIGURE 2:**
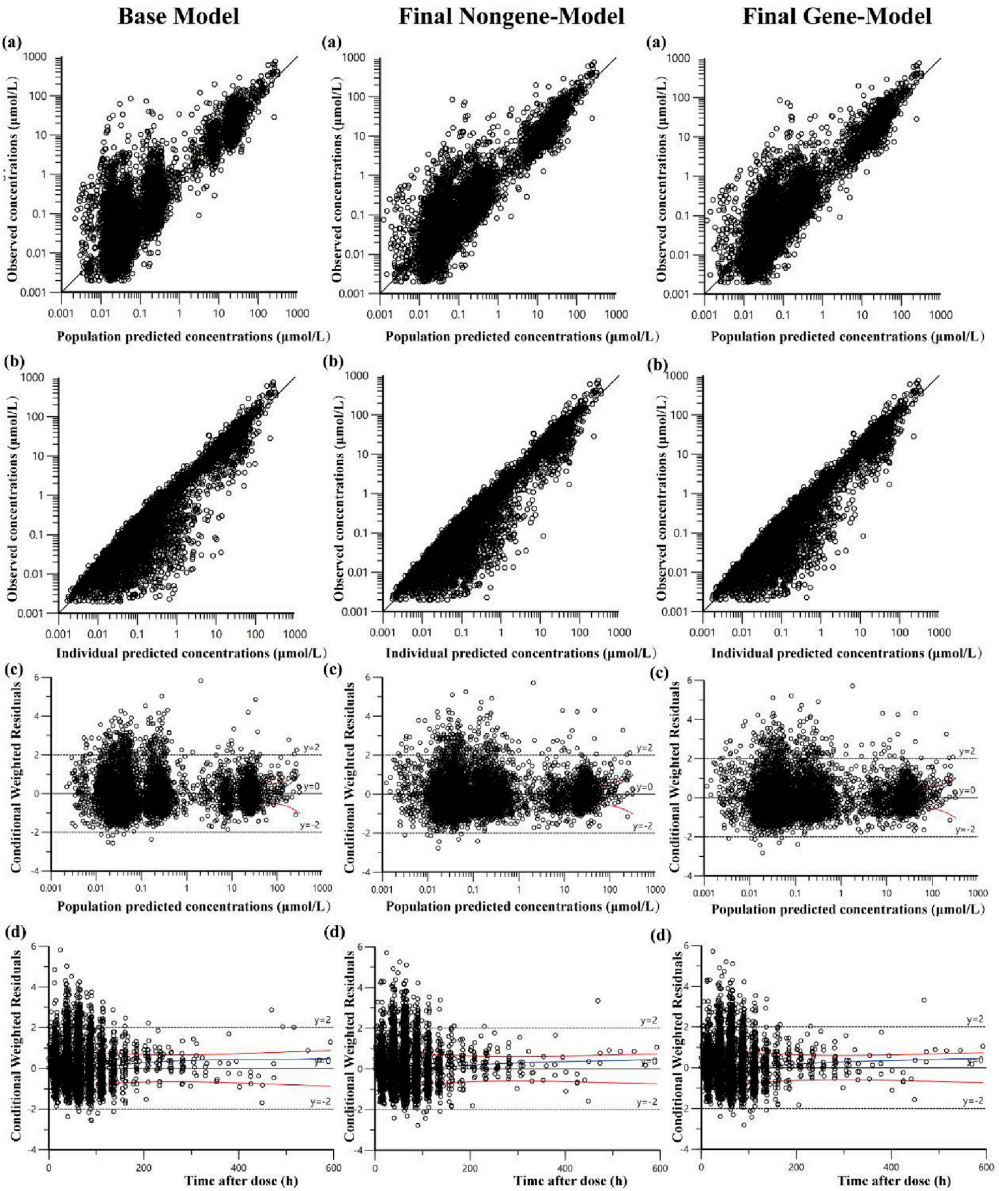
Diagnostic goodness-of fit plots of base model and final models: **(a)** observed versus population predicted concentrations (PRED); **(b)** observed versus individual predicted concentrations (IPRED); **(c)** conditional weighted residual (CWRES) versus PRED; **(d)** CWRES versus time after dose (TAD). In plots **(c,d)**, the two red lines represent the distribution of absolute CWRES values of the data and its mirror image, respectively.

The goodness-of-fit plots for the base and final models are presented in Figure 2. Scatter plots of observed concentrations versus PRED and IPRED (Figures 2A,B) demonstrate a strong correlation between predicted and observed values. However, in the plot comparing IPRED with observed values, the majority of data points, particularly at low concentrations, fell below the line of identity, suggesting a potential slight underestimation of CL. Nevertheless, this underestimation at low concentrations is unlikely to impact clinical decision-making. For example, concentrations below 0.05 show no significant difference in clinical implications. Comparison plots of CWRES versus PRED and TAD (Figures 2C,D) indicate that most residuals fall within two standard deviations and are evenly distributed around the axes. No significant biases are observed between CWRES and PRED or CWRES and time.

**FIGURE 3:**
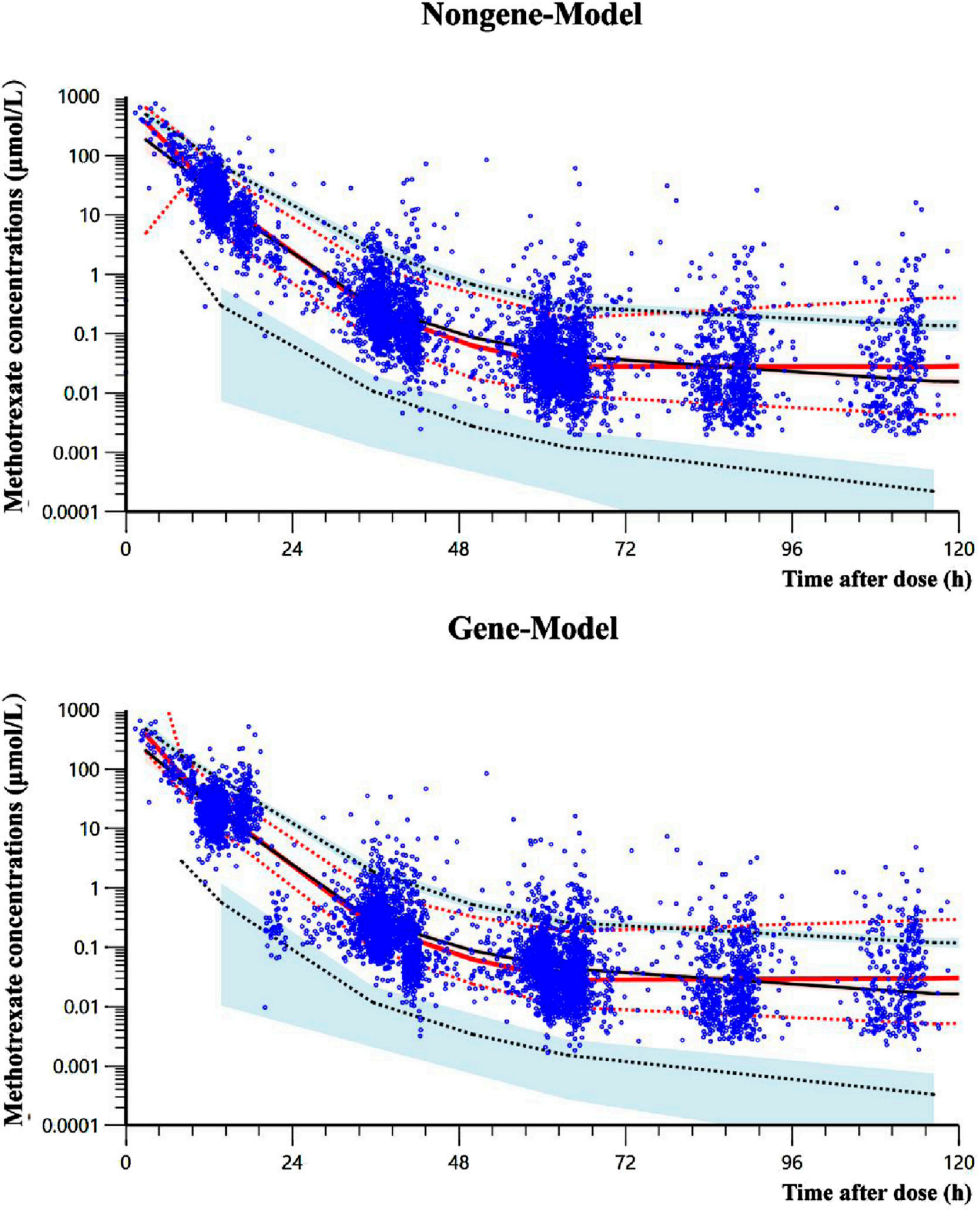
Visual predictive check results of two final models. The solid black line represents the median predicted concentration, while the two black dashed lines represent the 10th and 90th percentiles of the predicted concentration. The solid red line represents the median observed concentrations, while the two red dashed lines represent the 10th and 90th percentiles of the observed concentrations. The light red and light blue regions respectively represent the 95% confidence intervals for the median, 10th percentile and 90th percentile of the predicted concentrations. The observed data for methotrexate are denoted by blue dots.

In the bootstrap analysis of the final models, all 200 resampling iterations were successfully executed. The typical parameter estimates, standard errors, and 95% CI derived from the original dataset were consistent with the bootstrap results, as detailed in Table 5, indicating that the final models exhibit good stability and reproducibility. In the VPC analysis (Figure 3), the majority of observed concentrations were encompassed within the model’s 80% prediction intervals, with the median observed concentrations closely aligning with the median predicted values, demonstrating acceptable predictive performance of the models. However, due to the limited data available for concentrations beyond 120 h (only 5% [304/6,074] of measurements), the model’s ability to predict MTX concentrations may be insufficient when the time after dose exceeds 120 h (Supplementary Appendix SA4).

## 4 Discussion

This study provides the most comprehensive population pharmacokinetic assessment to date of HD-MTX in Chinese adult patients with PCNSL. The model supports time-specific estimation of plasma concentrations to identify delayed elimination and optimize leucovorin rescue strategies, while enabling AUC and Cmax calculations linked to MTX efficacy and toxicity. With further refinement, it may guide individualized dosing without prior concentration data. Although two-compartment models have been predominantly employed in prior studies to describe the pharmacokinetics of MTX, comparative model evaluation in our study demonstrated superior performance of the three-compartment model with first-order absorption and elimination (Joerger et al., 2006; Kim et al., 2012; Panetta et al., 2020; Batey et al., 2002; Faltaos et al., 2006; Fukuhara et al., 2008; Johansson et al., 2011; Zhang et al., 2015; Nader et al., 2017; Mei et al., 2018a; Hui et al., 2019; Kawakatsu et al., 2019; Yang L. et al., 2020; Aumente et al., 2006; Colom et al., 2009; Zhang et al., 2010; Faganel et al., 2011; Lui et al., 2018; Schulte et al., 2021; Beechinor et al., 2019; El Desoky et al., 2011; Gallais et al., 2020; Godfrey et al., 1998; Medellin-Garibay et al., 2020; Nagulu et al., 2010; Pai et al., 2020; Piard et al., 2007; Wright et al., 2015; Min et al., 2009; Yukawa et al., 2007; Zang et al., 2019). Specifically, the three-compartment model demonstrated a significantly improved fit to the observed data, as evidenced by a substantially lower OFV (17.69), AIC (41.69), and BIC (122.23) compared to the two-compartment model, which yielded an OFV of 807.46, AIC of 825.46, and BIC of 885.87. In previous studies on PPK models of HD-MTX in adult patients, the reported typical values and IIV ranges for MTX CL and Vc were 5.57–15.7 L/h (IIV, 17%–51.6%) and 19–79.2 L (IIV, 32.1%–48.3%), respectively (Zhang et al., 2022). In this study, the estimated CL (8.2 and 8.45 L/h, IIV: 27.3% and 27.1%) and Vc (33.39 and 33.29 L, IIV: 20.95% and 20.27%) for MTX were consistent with these findings. For peripheral compartment parameters, the estimated Q_1_ (0.04 L/h), Q_2_ (0.09 L/h), and V_p2_ (1.14 L) in this study were similar to those reported by Simon et al., who developed a three-compartment model (Q_1_ = 0.1 L/h, Q_2_ = 0.021 L/h, V_p1_ = 1.58 L, V_p2_ = 1.99 L), though our estimated V_p1_ (17.9 L) was slightly higher (Simon et al., 2013).

This study identified that eGFR, BUN, and ALT significantly influence influenced the clearance of MTX (*P* < 0.01). MTX is predominantly excreted through the kidneys, accounting for 80%–90% of the drug’s elimination. Renal function has been consistently identified as an independent predictor of MTX clearance (Green et al., 2006; Kawakatsu et al., 2019). Scr, BUN, eGFR, and CLcr are all critical indicators of renal function. Most research highlights the importance of CLcr in predicting MTX clearance (Joerger et al., 2006; Shi et al., 2020; Fukuhara et al., 2008; Simon et al., 2013; Hui et al., 2019; Yang L. et al., 2020; Wang et al., 2019; Godfrey et al., 1998). However, in this study, eGFR emerged as a superior predictor of drug clearance than CLcr and Scr. In line with previous findings, an increase in eGFR was associated with enhanced MTX clearance (Kim et al., 2012; Panetta et al., 2020; Kawakatsu et al., 2019). Additionally, this study identified a quantitative relationship between BUN levels and MTX clearance. BUN primarily serves as a marker for renal efficiency in eliminating protein metabolism byproducts, and the elevated BUN generally indicate compromised renal function or decreased plasma volume (Arihan et al., 2018). Our findings demonstrate that higher BUN are significantly linked to decreased MTX clearance, consistent with observations reported by de Oliveira Henz et al. (2023). Although BUN has previously been reported as a component in certain equations for eGFR and may thus be indirectly associated with MTX clearance, it was not incorporated into the eGFR calculation in our study (Millisor et al., 2017). The observed association between BUN and MTX clearance therefore appears to reflect an independent and complementary marker of renal function. This relationship is further supported by the low correlation between BUN and eGFR (R^2^ = 0.22), suggesting that the effect of BUN on MTX clearance is not attributable to collinearity with eGFR.

Approximately 10% of MTX is metabolized to 7-OH MTX mainly in the liver (Behera et al., 2014; Weigert et al., 2008). Elevated ALT are typically indicative of liver dysfunction. Previous studies have shown a significant correlation between ALT and MTX clearance, with some research reporting reductions in the Vc and Vp associated with increasing ALT (Kawakatsu et al., 2019; Dupuis et al., 2008). In our study, MTX clearance was minimally affected in patients with mild liver impairment. Conversely, in individuals with severe liver dysfunction (more than 5 to 20 times the upper limit of normal), MTX clearance increased by approximately 5%–9%. This increase may be attributed to impaired hepatic metabolism of MTX and the subsequent release of MTX from damaged liver cells, resulting in elevated systemic MTX concentrations and a slight increase in renal excretion. The exact mechanisms remain uncertain and necessitates further investigation through additional research.

MTX exhibits a protein binding rate of approximately 50%, indicating that TP, albumin, and globulin could impact its volume of distribution and clearance (Joerger et al., 2006). In our study, elevated TP levels were associated with a decrease in Q of MTX, which might increase protein bounded MTX and decrease unbound MTX, thereby decreased its transport into peripheral compartments. However, unlike the findings of Pai MP and Mao J, who reported a positive correlation between albumin and CL, this study did not observe such an association (Mao et al., 2022; Pai et al., 2020).

In addition, this study identified a significant association between MTX clearance and the combined polymorphisms of the *ABCC-ABCG-ADORA2A* gene variants, including *ABCC4* rs2274407 (T>G), *ABCG2* rs2231142 (G>T), and *ADORA2A* rs2298383 (C>T). Patients harboring the *ABCC-ABCG-ADORA2A* gene mutations demonstrated an approximately 9% reduction in MTX clearance. While this reduction may have limited clinical significance, it holds potential value in scientific research. The *ABCC4* encodes multidrug-resistant protein 4 (MRP4), an ATP-binding cassette C-subfamily transporter expressed in various tissues and cancers, and plays a key role in the pharmacokinetics and pharmacodynamics of multiple drugs (Wen et al., 2015; Wittgen et al., 2012). The rs2274407 (G912T; K304N) variant, situated at the 3′splice acceptor site of exon 8 in *ABCC4* pre-mRNA, has been shown not to affect MRP4 activity but may disrupt the normal splicing of *ABCC4* pre-mRNA (Mesrian Tanha et al., 2017). In our study, the T allele was associated with increased MTX clearance, suggesting reduced plasma concentrations in individuals with GT or TT genotypes, which may partly account for previously reported poorer 3-year disease-free survival among *ABCC4* rs2274407 T allele carriers with Pre-B cell acute lymphoblastic leukemia (P = 0.00019; OR, 13.17; 95% CI, 2.55–68.11) (Mesrian Tanha et al., 2017).

The *ABCG2* gene encodes breast cancer resistance protein (BCRP), a broadly expressed efflux transporter that limits substrate absorption and facilitates excretion (Hardwick et al., 2007; Kukal et al., 2021; Song et al., 2022). BCRP facilitates the transport of diverse agents, including antibiotics, antiepileptics, and chemotherapeutics, and is implicated in multidrug resistance (Hardwick et al., 2007; Kukal et al., 2021; Robey et al., 2007). The rs2231142 (Q141K) variant in exon 5 is among the most studied *ABCG2* polymorphisms. A meta-analysis showed that T allele carriers had a 1.5-fold increase in rosuvastatin exposure, reflected in higher AUC (lnGM, 0.43; 95% CI, 0.35–0.50; P < 0.00001) and Cmax (lnGM, 0.42; 95% CI, 0.33–0.51; P < 0.00001) (Song et al., 2022). Li et al. reported that rs2231142 was associated with reduced serum MTX concentrations, with patients carrying the GG genotype showing lower dose-normalized MTX levels at 24 h and a decreased proportion of high MTX levels (>0.5 μmol/L) at 42 h compared to GT/TT genotypes (P = 0.01 and 0.006, respectively) (Li et al., 2023). Consistent with prior studies, our research also links the T allele to reduced MTX CL, potentially due to impaired BCRP-mediated efflux altering MTX pharmacokinetics (Hegyi et al., 2017; Esmaili et al., 2020; Morisaki et al., 2005).

The rs2298383 variant in *ADORA2A*, located in a putative promoter region, has been associated with transcriptional regulation (Cannata et al., 2020). The CC genotype has been linked to increased leukoencephalopathy risk (P = 0.004; OR, 15.30; 95% CI, 2.43–96.60) (Tsujimoto et al., 2016). In this study, the C allele was associated with increased MTX clearance, possibly due to allele-specific differences in *ADORA2A* expression, as higher mRNA levels have been reported in CC genotype carriers (Tsujimoto et al., 2016).

Polymorphisms in additional genes related to the MTX pathway, such as *SLCO1B1, ABCC2, ABCB1,* and *MTHFR*, have been associated with MTX clearance in previous studies (Kim et al., 2012; Simon et al., 2013; Faganel et al., 2011; Lui et al., 2018; Schulte et al., 2021; Wang et al., 2019). For example, the *MTHFR* 677C>T variant is associated with decreased dihydrofolate reductase activity, resulting in increased MTX toxicity and reduced efficacy (Yang L. et al., 2020; Faganel et al., 2011). While some studies have reported a link between *MTHFR* and delayed MTX clearance, *MTHFR* is not directly involved in MTX metabolism or transport, and the underlying mechanism remains unclear (Faganel et al., 2011; Imanishi et al., 2007). *SLCO1B1*, expressed on the basolateral membrane of hepatocytes, regulates hepatic uptake of MTX and thus influences its pharmacokinetics (Schulte et al., 2021; Wang et al., 2019). However, in the present study, these genes did not significantly affect MTX parameters in the studied patients.

Previous research on MTX population pharmacokinetics has consistently identified body weight and body surface area as important covariates affecting MTX pharmacokinetic parameters, likely due to their association with basal metabolic rate and the size of organs involved in drug excretion (Panetta et al., 2020; Mei et al., 2018a; Hui et al., 2019; Taylor et al., 2020; Aumente et al., 2006; Faganel et al., 2011; Lui et al., 2018; Beechinor et al., 2019; Gallais et al., 2020; Medellin-Garibay et al., 2020; Odoul et al., 1999; Johnstone et al., 2005). Pai et al. proposed that vertebral height could influence MTX clearance in obese individuals, owing to its relationship with kidney size and function (Pai et al., 2020). Additionally, some studies have reported higher MTX clearance in males compared to females (Zhang et al., 2010; El Desoky et al., 2011; Godfrey et al., 1998). The concurrent use of PPIs in patients undergoing HD-MTX therapy significantly reduces the clearance of MTX and its metabolite 7-OH MTX, likely due to the inhibition of renal H^+^/K^+^-ATPase and interference with ATP-dependent MTX excretion via BCRP in the renal proximal tubules, resulting in elevated plasma MTX concentrations (Joerger et al., 2006; Bezabeh et al., 2012). However, our study did not find significant effects of these covariates on the model parameters. Furthermore, factors such as pre-dose alkalinization, urine output/hydration status, and concomitant use of medications, including penicillin, and vancomycin, have been suggested to potentially affect MTX clearance (Joerger et al., 2006; Kim et al., 2012; Panetta et al., 2020; Batey et al., 2002; Zhang et al., 2015; Hui et al., 2019; Kawakatsu et al., 2019; Isono et al., 2021; Zhang et al., 2010). Due to the absence of data on these factors, a detailed analysis was not possible.

This study has several limitations: (1) The limited sample size during the distribution phase may introduce bias in the estimation of Vp and Q. (2) The concentrations of MTX in urine were not assessed, preventing accurate calculation of inter-compartmental clearance. (3) Data on variables such as urine output, urine pH and renal replacement therapy, which may significantly impact MTX clearance, were available for only a limited number of patients. This constraint precludes a comprehensive assessment of their effects on MTX clearance and may limit the overall completeness of the study’s findings. (4) Due to limitations in genotyping technology, not all relevant genetic variants were analyzed, and some important genes may have been overlooked. (5) The lack of data from other centers for patients with PCNSL restricts the external validation of the model. (6) The model’s predictive accuracy for MTX concentrations may be limited beyond 120 h due to the scarcity of data at these extended time points. (7) Although the lack of statistical significance for drugs with potential interactions, such as omeprazole and NSAIDs, was observed, caution is still warranted in decision-making.

## 5 Conclusion

Two PPK models have been successfully developed for HD-MTX in Chinese adult patients with PCNSL. In these models, MTX clearance decreased with lower eGFR, reduced ALT, elevated BUN, and the presence of *ABCC-ABCG-ADORA2A* mutations, while the inter-compartment clearance of MTX decreased with higher TP. Both models demonstrated stability and satisfactory predictive performance, showing potential to facilitate individualized MTX therapy for patients with PCNSL in the future.

## Data Availability

The original contributions presented in the study are included in the article/Supplementary Material, further inquiries can be directed to the corresponding authors.
