# Supplementary material for: Population pharmacokinetics of high-dose methotrexate in patients with primary central nervous system lymphoma
Source: Front Pharmacol. 2025 May 19;16:1578033. doi: 10.3389/fphar.2025.1578033 (PMC12127635; doi:10.3389/fphar.2025.1578033)
Supplement: Supplementary file 1 [file DataSheet1.docx]

**Appendix 1. Selected variants for methotrexate of enrolled patients**

| No. | Gene | SNP ID | Position | Variation | Residue change | Function | Genotype | MAF | HWE  (*P*-value) |
| --- | --- | --- | --- | --- | --- | --- | --- | --- | --- |
| 1 | ABCB1 | rs1045642 | exon | G>A |  | synonymous variant | 115/346/282 | 0.3876 | 0.5895 |
| 2 | ABCB1 | rs1128503 | exon | A>G |  | synonymous variant | 97/338/315 | 0.3547 | 0.69 |
| 3 | ABCC2 | rs3740065 | upstream transcript | A>G |  |  | 106/335/307 | 0.3656 | 0.3448 |
| 4 | ABCC2 | rs717620 | upstream transcript | C>T |  |  | 34/261/455 | 0.2193 | 0.749 |
| 5 | ABCC2 | rs2273697 | exon | G>A | Val417Ile | missense | 6/112/443 | 0.1105 | 1 |
| 6 | ABCC4 | rs2274407 | exon | C>A | Lys304Asn; | missense | 17/173/560 | 0.138 | 0.4412 |
| 7 | ABCG2 | rs2231142 | exon | G>T | Gln141Lys | missense | 68/321/360 | 0.3051 | 0.7966 |
| 8 | ADORA2A | rs2298383 | upstream transcript | C>T |  |  | 179/383/187 | 0.4947 | 0.5592 |
| 9 | ATIC | rs2372536 | exon | C>G | Thr116Ser | missense | 49/283/419 | 0.2537 | 0.9232 |
| 10 | ATIC | rs7563206 | intron | C>T |  |  | 43/275/431 | 0.241 | 1 |
| 11 | ATIC | rs12995526 | intron | C>T |  |  | 44/276/429 | 0.243 | 1 |
| 12 | ATIC | rs4673993 | intron | T>C |  |  | 53/283/407 | 0.2618 | 0.7042 |
| 13 | CCND1 | rs9344 | exon | A>G |  | synonymous variant | 168/385/196 | 0.4813 | 0.4642 |
| 14 | FPGS | rs10106 | intron | C>T |  | intron | 92/345/313 | 0.3527 | 0.8731 |
| 15 | FPGS | rs10760502 | exon | G>A | Ile22Val | missense | 14/41/521 | 0.0599 | <0.001 |
| 16 | GGH | rs3758149 | upstream transcript | G>A |  |  | 57/173/515 | 0.1926 | <0.001 |
| 17 | GSTP1 | rs1695 | exon | A>G | Ile105Val | missense | 24/237/489 | 0.19 | 0.5528 |
| 18 | MTHFD1 | rs2236225 | exon | G>A | Arg653Gln | missense | 39/282/425 | 0.2413 | 0.4239 |
| 19 | MTHFR | rs1801131 | exon | T>G | Glu470Ala | missense | 14/210/525 | 0.1589 | 0.2182 |
| 20 | MTHFR | rs1801133 | exon | A>G | Ala263Val | missense | 176/350/220 | 0.4705 | 0.1226 |
| 21 | MTHFR | rs2274976 | exon | C>T | Arg635Gln | missense | 3/74/670 | 0.05355 | 0.463 |
| 22 | MTR | rs1805087 | exon | A>G | Asp975Gly | missense | 5/124/623 | 0.0891 | 0.8237 |
| 23 | MTRR | rs1801394 | exon | A>G | Ile37Met | missense | 44/280/413 | 0.2497 | 0.7682 |
| 24 | NOS3 | rs1799983 | exon | G>T | Asp298Glu | missense | 10/128/612 | 0.09867 | 0.3 |
| 25 | SLC19A1 | rs1051266 | exon | C>T | His27Arg | missense | 141/313/197 | 0.457 | 0.4302 |
| 26 | SLC28A2 | rs2413775 | intron | T>A |  |  | 18/254/476 | 0.1939 | 0.01898 |
| 27 | SLCO1B1 | rs4149081 | intron | G>A |  |  | 123/333/296 | 0.385 | 0.07669 |
| 28 | SLCO1B1 | rs11045879 | intron | T>C |  |  | 123/282/315 | 0.3667 | <0.001 |
| 29 | TYMS | rs151264360 | exon | D>I |  | cds indel, downstream variant 500B, intron variant, utr variant 3 prime | 80/339/329 | 0.3336 | 0.6225 |

**Appendix 2. Plot of methotrexate (MTX) plasma concentration vs time**


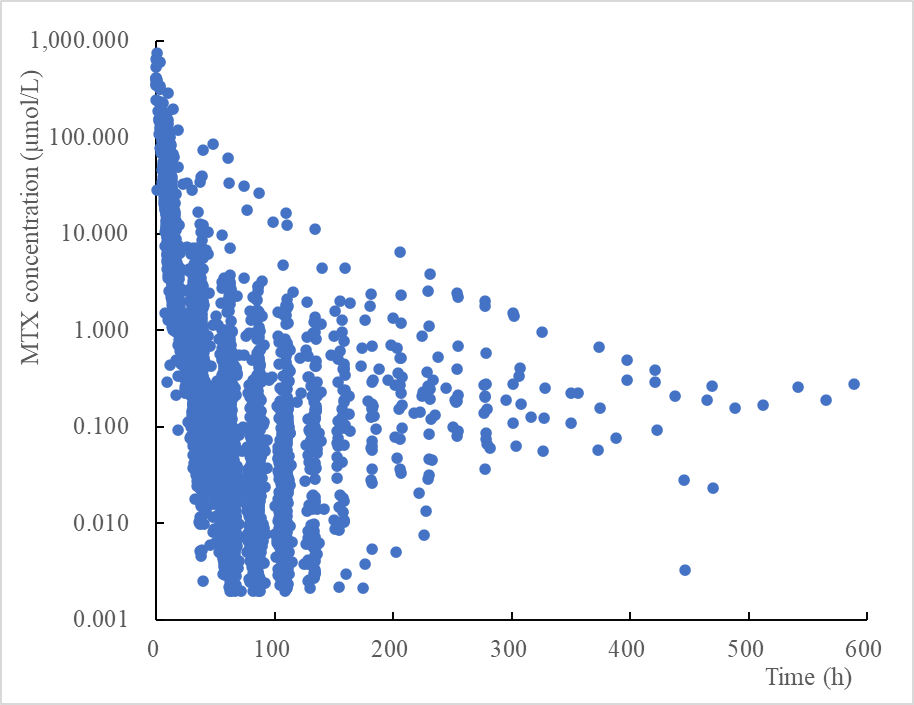


**Appendix 3. Plot of Estimated glomerular filtration rate vs time**


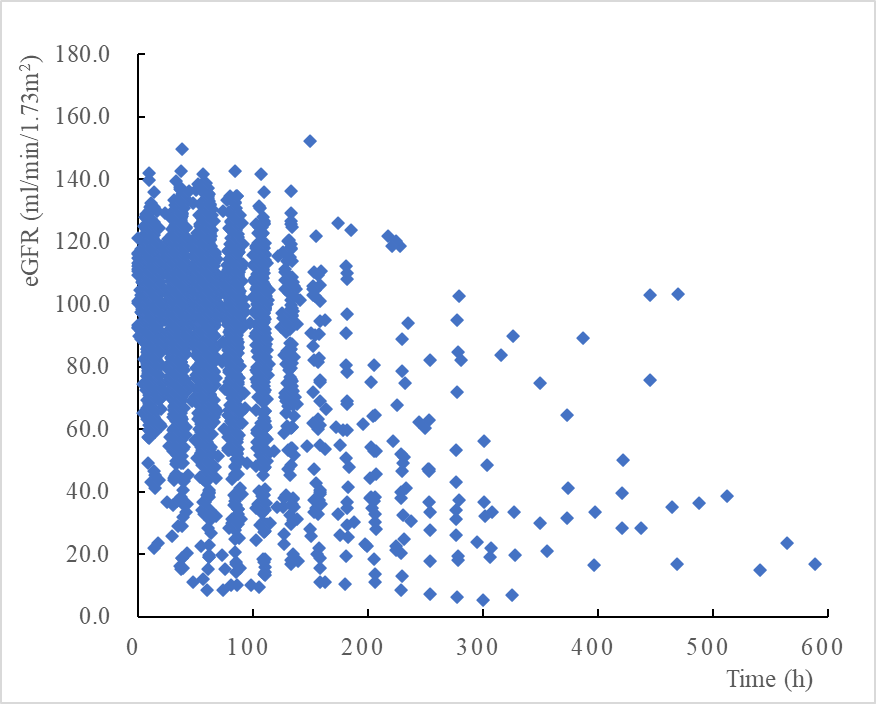


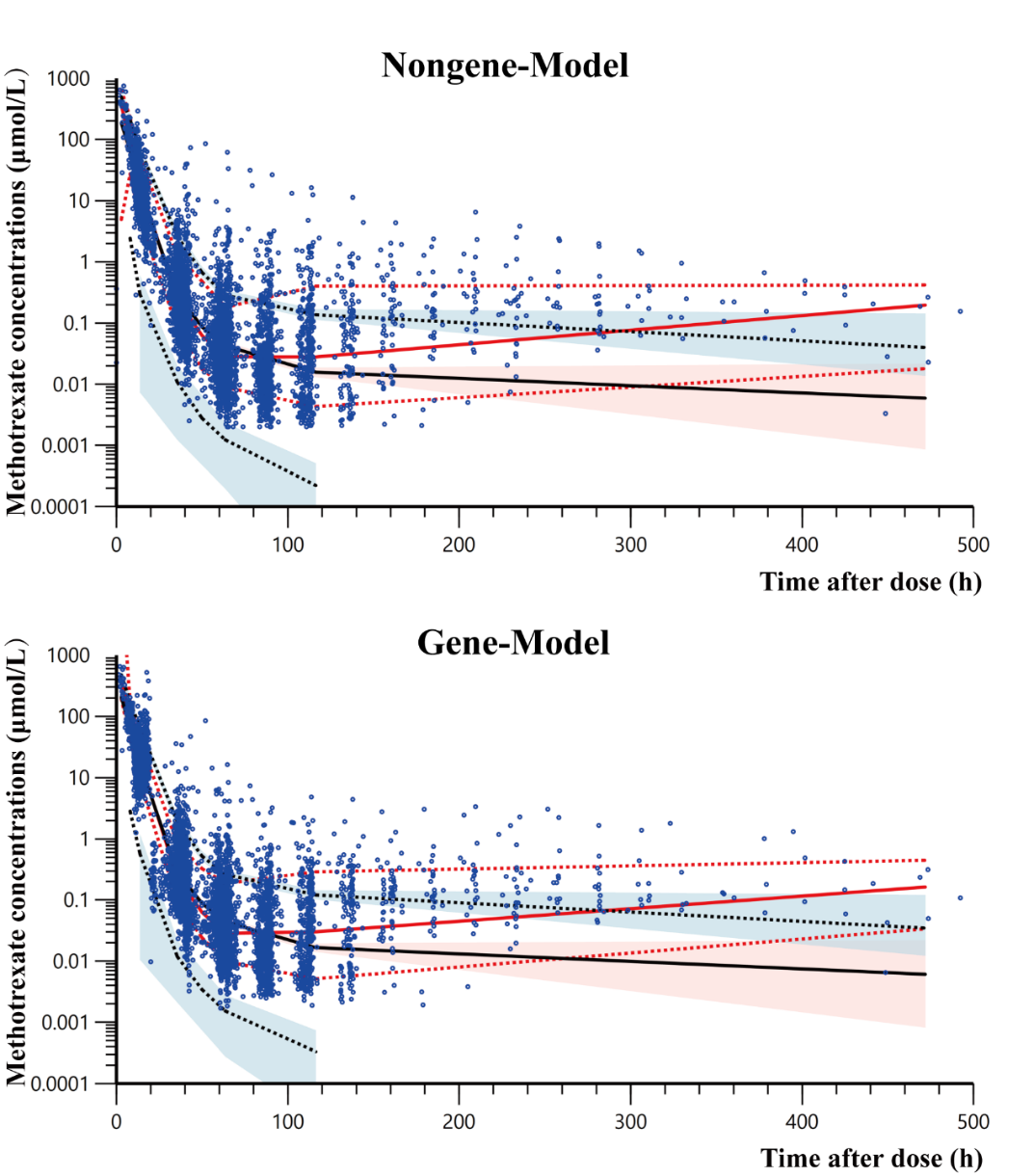
**Appendix 4. Visual predictive check results of two final models**

The solid black line represents the median predicted concentration, while the two black dashed lines represent the 10th and 90th percentiles of the predicted concentration. The solid red line represents the median observed concentrations, while the two red dashed lines represent the 10th and 90th percentiles of the observed concentrations. The light red and light blue regions respectively represent the 95% confidence intervals for the median, 10th percentile and 90th percentile of the predicted concentrations. The observed data for methotrexate are denoted by blue dots.
